# Epidemiological study on Ixodid tick infestation and tick borne haemopathogens on cattle in Awi Zone, northwest Ethiopia

**DOI:** 10.1002/vms3.878

**Published:** 2022-07-08

**Authors:** Hailemariam Adugna, Habtamu Tamrat

**Affiliations:** ^1^ Awi zone Livestock Resource Development Office Animal Health Department Injibara Ethiopia; ^2^ School of Animal science and Veterinary Medicine College of Agriculture and Environmental Science, Bahir Dar University Bahir Dar, Ethiopia

**Keywords:** cattle, districts, Ethiopia, haemoparasite, Ixodid tick, prevalence, risk factors

## Abstract

**Background:**

Tick and tick borne haemopathogens are the main challenge of livestock production and productivity in Ethiopia particular in northwest Ethiopia due to favourable climate condition.

**Objectives:**

The objectives of this study was to determining the prevalence of Ixodid tick infestation and tick borne haemopathogens on cattle, identifying the existing Ixodid tick species, assessing seasonal variation and major risk factors associated with tick infestation and tick borne haemopathogens.

**Methods:**

A cross‐sectional study was conducted from December 2020 to july 2021 on Ixodid tick infestation and tick borne haemopathogens on cattle in the northwest Ethiopia. A toatal of 384 cattle were used for this study. Tick species were identified using morphological identification keys under a stereomicroscope. Thin blood smear examination were conducted to assess tick borne haemopathogens.

**Results:**

The overall prevalence of Ixodid tick infestation and tick borne haemopathogens were 45% and 3%, respectively. *Babesia bigemina* was the only haemoparasite detected in the present survey. Potential risk factors were investigated for their association with tick infestation and *B. bigemina* using logistic regression and chi‐square test, respectively. Accordingly, age, body condition, agroecological systems and season were significantly (*p* < 0.05) associated with tick infestation whereas season and body condition were significantly (*p* < 0.05) associated with *B. bigemina* infection. A total seven tick species were identified. *Amblyomma varigatum* (55%) and *Boophilus decoloratus* (15.7%) were the predominant Ixodid tick species encountered. A total of 128 blood samples were collected from Jawi district and examined using thin blood smear. Of them, 3% were infected by the *B. bigemina*

**Conclusions:**

Tick infestation in this study was high and seems to play vital role for the reduction of production, productivity and for the transmission *B. bigemina*. Therefore, sound and effective tick control and prevention strategies are needed to mitigate the risk factors for tick infestation and *B. bigemina* infection in Ethiopia.

## Graphical abstract

A cross‐sectional study was conducted from December 2020 to July 2021 on Ixodid tick infestation and tick borne haemopathogens in North West Ethiopia. The overall prevalence of Ixodid tick infestation and tick borne haemopathogens were 45% and 3%, respectively. Babesia bigemina was the only haemoparasite detected in the present survey. Age, body condition, agroecological systems and season were significantly (P<0.05) associated with tick infestation whereas season and body condition were significantly (P<0.05) associated with B. bigemina infection. A total seven tick species were identified. Amblyomma varigatum (55%) and Boophilus decoloratus (15.7%) were the predominant Ixodid tick species encountered. A total of 128 blood samples were collected from jawi district and examined using thin blood smear. Of them, 3% were infected by the B.bigemina. Tick infestation in this study was high and seems to play vital role for the reduction of production, productivity, and for the transmission B.bigemina. Therefore, sound and effective tick control and prevention strategies are needed to mitigate the risk factors for tick infestation and B. bigemina infection in Ethiopia.

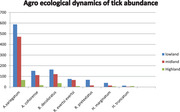

## 1 INTRODUCTION

Ethiopia has an extremely diverse topography, a variety climatic features and agroecological zones that are expedient to host a very large animal population (Mekasha & Zewdie, 2014). Ethiopia is one of the countries with the largest number of livestock population in Africa (Alekaw, 2000). The country has the estimated domestic animal number of 65.35 million cattle, 39.89 million sheep, 50.5 million goats, 7.7 million camels, 2.11 million horses, 0.38 million mules, 8.98 million donkeys and 48.96 million poultry (CSA, 2019/2020). The livestock subsector has an enormous contribution to Ethiopia's national economy and livelihoods of many Ethiopians. The subsector contributes about 16.5% of the national Gross Domestic Product (GDP) and 35.6% of the agricultural Gross Domestic Product (GDP). Livestock mainly cattle in Ethiopia represent the pillar of the economy and plays vital roles in the socioeconomic aspects of the life of the people (Metaferia et al., 2011).

Despite the largest livestock population in Ethiopia, the economic benefits remain marginal due to prevailing diseases, poor management and low genetic performance (Dabassa et al., 2017; Jilo et al., 2017). In Ethiopia, ticks occupy the first place among the external parasites through mortality of animals, decreased production, downgrading and general rejection of skins and hides (Dabassa et al., 2017). The impacts of ticks on animals were either by inflecting direct damage or by transmission of tick‐borne pathogens. They are responsible for severe economic losses both through the direct effects associated with their blood sucking behaviour (Kumsa et al., 2015a) and also indirectly act as reservoirs and vectors for a wide range of human and animal pathogens (Jongejan & Uilenberg, 2004). Ticks and tick‐borne diseases affect 90% of the world's cattle population and are widely distributed throughout the world (Estrada‐Peña et al., 2014). The country's environmental condition and vegetation are highly conducive for ticks and tick‐borne disease perpetuation. The presence of diseases caused by haemoparasites is broadly related to the presence and distribution of their vectors. Ticks are more prevalent in the warmer climates, especially in tropical and sub‐tropical areas (Ikpeze et al., 2015). Previous study conducted in different part of Ethiopia revealed that there are five genera and forty seven species of Ixodid ticks found on livestock (Hailu et al., 2008; Kumsa et al., 2016; Pegram et al., 2004; Tadesse & Sultan, 2014).

Tick borne haemopathogen have a serious economic impact on livestock sector due to decreased productivity, lowered working efficiency, increased cost for control measures and limiting introduction of genetically improved cattle in the area and death of livestock (Radostits et al., 2008; Uilenberg, 1995). In Ethiopia, anaplasmosis, babesoisis, cowdrosis and theileriosis have been reported as major tick borne diseases affecting domestic animals (Teshale et al., 2016). Among tick‐borne pathogens of zoonotic importance spotted fever, *Borrelia* spp., *Coxiella burnetii* and *Bartonella* spp. have been documented from Ethiopia (Kumsa et al., 2015a).

There are different ways of classifying the climatic systems of Ethiopia, including the traditional, the Köppen's, the Throthwaite's, the rainfall regimes and the agroclimatic zone classification systems (Yohannes, 2003). The most commonly used classification systems are the traditional and the agroecological zones. According to the traditional classification system, which mainly relies on altitude and temperature, Ethiopia has five climatic zones such as ‘Wurch’ (upper highland), ‘Dega’ (highland), ‘Weyna dega’ (midland), ‘kola’ (lowland) and ‘Berha’ (Desert) (MoA, 2000). The seasonal variations within a bioclimatic zone may favour or hinder the development or activity of a tick species during certain periods (Latif & Walker, 2004). Dry environmental conditions are a serious danger to ticks, particularly to the questing larvae, which are very susceptible to drying out fatally (Walker et al., 2014). Also, the start and end of the rainy season may influence the different phases of the life cycle (Getachew et al., 2014). Other species are more restricted to a specific habitat where their specific hosts are present and where climatic conditions allow survival and reproduction (Mekonnen et al., 2007).

Although quite a lot of similar studies on Ixodid tick infestation and tick borne haemopathogen in cattle have been conducted in different areas of Ethiopia, it is worth noting that Ethiopia is a large country with a huge number of livestock populations, mostly cattle, and therefore most of the studies are targeting only specific areas, and not the whole country. Furthermore, there was no known research conducted in the past and no any published information regarding Ixodid tick infestation and tick borne haemopathogen in cattle in the study area. Therefore, the objectives of this study were aimed to determine the prevalence of Ixodid tick infestation and tick borne haemopathogen, to identify the existing Ixodid tick species, to assess seasonal and agroecological variations of Ixodid ticks and to investigate the major risk factors associated with tick infestation and tick borne haemopathogen in the study area.

## 2 MATERIALS AND METHODS

### 2.1 Description of the study areas

The study was conducted in two selected districts of Awi Zone, Amhara National Regional State, northwest Ethiopia. Awi Zone is located within the latitude of 10.95°N and longitude of 36.5°E. It lies at an altitude range of 648–3100 m above sea level (m a.s.l.) with average altitude of 2300 (m a.s.l) (Yeshambel et al., 2011). It is predominantly inhabited by the Awi, ethnic group which belongs to the central cushitic subfamily and inhabits in northwest Ethiopia (Alamneh, 2004). The livestock population in the district includes 1,204,367 cattle, 377,727 sheep, 190,873 goats, 70,012 horses, 97,749 donkeys and 954,973 poultry (AAZLDO, 2020).

### 2.2 Ankasha district

Ankasha Guagusa Woreda is located in Awi zone in the northwestern part of Ethiopia, approximately 480 km northwest of Addis Ababa, the capital city and 120 km southwest of Bahir Dar, capital of the Amhara region. The absolute location ranges between the coordinates of 10°31′46’’ and 10°41′32’’ north latitude and 36°36′18’’ and 36°59′33’’ east longitude. The district is covered by the vegetation type dry Afromontane forest, which is dominated by *Albizia gummifera*, macrostachyus and *Apodytes dimidiate*. This district receives 2057.5 mm mean annual rainfall. The highest amount of rainfall is recorded in July and August that reaches above 450 mm per month at the peak period. This woreda has an elevation ranging from 1200 to 2800 m a.s.l. The annual mean temperature of this area is 14–32°C. The weather condition provides the highlands with most of its rainfall during a period that generally lasts from mid‐June to mid‐November. Here, animal husbandry is mainly extensive. This woreda has a total 166,821 cattle, 63,965 sheep, 21,432 goats, 12,453 horses and 121,986 poultry (AGWLDO, 2020).

### 2.3 Jawi district

Jawi district is found in Awi zone, Amhara regional state, northwest Ethiopia. The climate alternates with long summer rainfall (June to September) and a winter dry season (October to May) with mean annual rain fall of 1569.4 mm. The annual mean temperature varies between 16.68°C and 37.6°C and the altitude ranges from 648–1300 m a.s.1. The land is covered by different vegetation types namely savanna grassland, forest, riverine and bush lands with major agricultural products like sorghum, maize, sesame and cotton. Jawi woreda comprises about 213,642 cattle, 20,649 sheep, 64,846 goats, 53 equines and 62,958 poultry (AAZLDO, 2020).

### 2.4 Study population

The study population were local/zebu cattle breed kept under individual households with different age, sex and body condition scores found in the two selected districts. The animals are managed with extensive management system and depend on grazing throughout the year for their feed sources with little supplementation of crop residues.

### 2.5 Study design and sampling procedure

A cross sectional study was conducted from December 2020 to July 2021 to determine epidemiology of Ixodid tick infestation and tick borne haemoparasites on Awi Zone, northwest Ethiopia. For this study, two districts were selected purposely based on the agroecological systems, which favours tick infestation while the study animals were selected using simple random sampling. Jawi district has a lowland agroecological systems ranges from 648–1300 m a.s.l. Ankasha district has both midland and highland agroecologies ranges from 1200 to 2800 m a.s.l. Multistage sampling was used to select kebeles and household levels. In multistage sampling, the two districts are primary units and in districts kebeles were selected randomly which were considered as secondary units. Within kebeles households were selected randomly and considered as tertiary unit. ‘Kebeles’ corresponds to the smaller territorial administrative unit, which is a tight system of neighbourhood administration or a collection of peasant associations and households. A group of kebeles form a district. Information about agroecological systems and proportionally equal sample were taken in dry and wet season. The study animals were classified into two groups based on age as young (≤2 years) and adult (>2 years) according to Okello‐Onen et al. (1999) and body condition score was recorded after classifying the animals into poor, medium and good (Verhees et al., 2011).

### 2.6 Sample size determination

The sample size required for this study was determined according to Thrusfield (2005). Since there is no documented information about on Ixodid tick infestation and tick borne haemoparasites on cattle in study area, assuming a 50% of expected prevalence, at 95% of confidence interval and 5% of required absolute precision was used. Thus, a total of 384 indigenous zebu cattle, 128 from Jawi (lowland) district and 256 cattle in Ankasha district (128 cattle in highland and 128 cattle in midland), were used for this study.

### 2.7 Data collection

#### 2.7.1 Tick sample collection

Tick were collected after casted down and restrained appropriately. Then, the skin of each selected cattle was inspected for the presence or absence of ticks from half body part. The favourable predilection anatomical sites where ticks were searched are scrotum/udder, Groin, dewlap, belly, tail, leg/hoof and neck. All visible adult ticks were manually collected by using forceps from half regions of the animals’ body and care was taken to avoid decapitulation (Walker et al., 2014). The collected tick was placed in a labelled clean universal bottle using 70% alcohol and transported to Bahir Dar regional animal health disease investigation and diagnostic laboratory centre for tick species identification.

#### 2.7.2 Blood sample collection

For blood sample collection, Jawi district was purposely selected due to financial limitation and a total of 128 blood samples were collected from ear vein using heparinised capillary tube and sealed from randomly selected cattle from Jawi district (lowland agroecological systems) following the standard protocol described by Urquhart et al. (1996). After labelling, it was kept in cold chain and examined at Jawi Woreda veterinary clinic laboratory by thin blood smear technique. All of 128 blood samples were examined by microscope at ×100 magnification power.

### 2.8 Parasitological examination

#### 2.8.1 Tick identification

Ticks were identified to the species level according to their morphological key structures such as shape of scutum, leg colour, scutum ornamentation, body grooves, punctuations, basis capitulum, coaxes and ventral plates. During tick identification in the laboratory, the sample was put on Petri dish and adult ticks were identified to species level under a stereomicroscope using the standard identification keys (Houseman, 2013; Walker et al., 2014).

#### 2.8.2 Haemoparasite examination

**Thin blood smear**: Giemsa staining procedures and microscopic examinations were conducted according to OIE (2010) and Zafar et al. (2006). Each stained slides were examined for identification of blood protozoa (Urquhart et al., 1996). The parasite was identified by the characters described by Soulsby (1982). The numbers of microscopic fields, which were examined per slide for the detecting haemoparasites in the sampled cattle, were six microscopic fields from one positive animal, two microscopic fields from the two positive samples and one microscopic field from one positive sample. However, after examination of the whole parts of the prepared microscopic slide and if we do not get any haemoparasites, an animal is negative.

#### 2.8.3 Data management and analysis

All data collected from laboratory examination were organised and feed into Microsoft Excel spread sheets and coded appropriately and analysed using STATA version 14.0 statistical software. The data were summarised by descriptive statistics and displayed by tables and graphs. Poisson regression was used to analyse the number (count) of ticks on animals as a function of different explanatory variables (season and agroecological systems). Logistic regression was employed to analyse the degree of association of binary outcome (presence or absence of tick) as a function of various potential factors and to measure the strength of the associations. Odds ratio and chi‐square tests were used to quantify the association among the factors with the presence of tick infestation and *B. bigemina*, respectively. Effects were reported as statistically significant in all cases if value is less than 5% at 95% confidence interval. We have considered for the risk factor analysis the lowland and midland agroecological systems, and the lowland agroecological system for the Ankasha and Jawi districts, respectively. Age, sex, body score condition and season were also considered in both districts for this analysis. Prevalence indicators were calculated for ticks and tick‐borne pathogens as follows: Tick infestation prevalence = number of cattle infested by ticks/total number of surveyed cattle (384). Tick‐borne pathogens infection prevalence = number of cattle with positive blood smears to haemopathogens/total number of surveyed cattle.

## 3 RESULTS

### 3.1 Prevalence of Ixodid tick infestation and tick borne haemoparasite

A total of 384 cattle were examined for tick infestation. Of which, 173 cattle were found positive for tick infestation with overall prevalence of 45%. In the current study, 128 blood samples were collected from Jawi district for examining the presence of tick borne haemoparasite in both wet and dry season. Of them, four cattle were positive for *B. bigemina* with overall prevalence of 3% (Table 1). The parasitaemia levels of *B. bigemina* were low and medium from the three and one positive sampled animals, respectively.

**TABLE 1:** Overall prevalence of Ixodid tick infestation and *Babesia bigemina* in the study area

Districts	*N*	Number positive	Number positive for haemoparasite	Prevalence of tick infestation (%)	Prevalence of tick borne haemoparasite (%)	OR	95% CI	*p* Value
Ankasha*	256	99		38.7				
Jawi	128	74	4	57.8	3	2.17	1.41–3.34	0.000
Overall	384	173	4	45	3			

* Reference variable, *N* = number of sampled animal.

### 3.2 Association of potential risk factors with Ixodid tick infestation

A total of five potential risk factors were tested using multivariate logistic regression Accordingly, age, body condition, agroecological systems and season were statistically significant (*p* < 0.05) associated with tick infestation in the districts. Keeping the effect of other variables constant, the odd of tick infestation in younger aged cattle was 1.94 times more likely to be infested than their older counter parts. Similarly, the odd of tick infestation in wet season was 15.65 times higher than dry season. The association between body condition and agroecological systems with tick infestation were indicated (Table 2).

**TABLE 2:** Potential risk factors significantly associated to cattle tick infestation using multivariate logistic regression analysis

Risk factors	*N*	No. of animals infested	Prevalence (%)	OR	95% CI	*p* Value
Age						
Young*	77	26	33.8		1.01–3.72	0.044
Adult	307	147	47.9	1.94		
Sex						
Female*	250	120	48		0.35–1.05	0.077
Male	134	53	39.5	0.61		
BCS						
Good*	68	19	27.9		1.78–4.36	0.000
Medium	237	107	45.1	2.78		
Poor	79	47	59.5			
Agroecology						
Highland*	128	38	29.7		1.96–3.93	0.000
Midland	128	61	47.7	2.77		
Lowland	128	74	57.8			
Season						
Dry*	192	37	19.3		8.86–27.65	0.000
Wet	192	136	70.8	15.65		

* Reference variable, *N* = number of sampled animal.

BCS, body condition score.

### 3.3 Association of potential risk factors with tick borne haemoparasites

A total of four potential risk factors were tested using chi‐square test (*X* ^2^). Accordingly, body condition and season were statistically significant (*p* < 0.05) associated with tick born haemoparasite. Keeping the effect of other variables constant, the odd of tick born haemoparasite in poor conditioned cattle was 11.9 times higher than well‐conditioned cattle. The statistical significance of association between seasons and tick‐borne haemoparasite is indicated in (Table 3).

**TABLE 3:** Prevalence of *Babesia bigemina* and association with different risk factors by chi‐square test (χ^2^)

Risk factor categories	*N*	No. of animals infected	Prevalence (%)	Chi‐square ( χ^2^)	*p* Value
Age					
Young	27	0	0	1.1038	0.293
Adult	101	4	4		
Sex					
Male	43	1	2.3	0.13	0.712
Female	85	3	3.5		
BCS					
Poor	19	3	15	11.9	0.03
Medium	84	1	1		
Good	25	0	0		
Season					
Dry	64	0	0	4.129	0.042
Wet	64	4	6		

*N*, number of sampled animal.

BCS, body condition score.

### 3.4 Identification of Ixodid tick species

A total of 2047 (1439 males and 608 females) ticks were collected in the districts. In the current study, four tick's genera and seven tick species were identified. *Amblyomma* and *Hyalomma* were the predominant and the lowest tick genera with prevalence of 68.7% and 4.1%, respectively (Table 4). The prevalence of other tick genera by season and agroecological systems was indicated (Tables 5 and 7), respectively. Similarly, *A. variegatum* was the predominant tick species while *H. trucantum* was the lowest tick species in the area (Table 6).

**TABLE 4:** Poisson regression analysis of significant value ticks’ genera collected from the three agroecological systems

	Agroecological systems			
Tick genera	Lowland	Midland	Highland	Overall count (%)	*p* value
Amblyomma	740 (67.3%)	584 (72.6%)	83 (58%)	1407 (68.7%)	0.000
Boophilus	164 (14.9%)	121 (15%)	36 (25.2%)	321 (15.7%)	0.000
Rhipicephalus	144 (13.1%)	81 (10.1%)	9 (6.3%)	234 (11.4%)	0.000
Hyalomma	52 (47.3%)	18 (2.2%)	15 (10.5%)	85 (4.1%)	0.000
Total count	1100 (53.8%)	804 (39.2%)	143 (7%)	2047 (100%)	0.000

**TABLE 5:** Poisson regression analysis of significant value of ticks’ genera collected in dry and wet seasons

Tick genera	Season			
	Wet	Dry	Over all count	*p* value
Amblyomma	1279 (70.9%)	128 (52.9%)	1407 (68.7%)	0.000
Boophilus	268 (14.8%)	53 (2.2%)	321 (15.7%)	0.000
Rhipicephalus	200 (11.1%)	34 (1.4%)	234 (11.4%)	0.000
Hyalomma	58 (3.2%)	27 (1.1%)	85 (4.2%)	0.000
Total count	1805 (88.2%)	242 (11.8%)	2047 (100%)	0.000

**TABLE 6:** Diversity, count and percentages of tick species on cattle during the wet and dry seasons in three agroecological systems

	Agroecological systems									
	Lowland			Midland			Highland			
Tick species	Wet count (%)	Dry count (%)	Total count (%)	Wet count (%)	Dry count (%)	Total count (%)	Wet count (%)	Dry count (%)	Total count (%)	Overall count (%)
A. v	542 (56.2)	46 (33.8)	588 (53.5)	435 (60.2)	37 (45.7)	472 (58.7)	55 (46.6)	11 (44)	66 (46.2)	1126 (55)
A. c	131 (13.6)	21 (15.4)	152 (13.9)	99 (13.7)	13 (16)	112 (14)	17 (14.4)	0 (0)	17 (1.9)	281 (13.7)
B. d	129 (13.4)	35 (25.7)	164 (15)	110 (15.2)	11 (13.6)	121 (15)	29 (24.6)	7 (2.8)	36 (25.2)	321 (15.7)
R. e	69 (7.2%)	7 (5.2)	76 (7)	57 (7.9)	9 (0.1)	66 (8.2)	5 (4.2)	4 (1.6)	9 (6.3)	151 (7.4)
R. p	56 (5.8)	12 (8.8)	68 (6.1)	13 (0.2)	2 (0.03)	15 (1.9)	0 (0)	0 (0)	0 (0)	83 (4.1)
H. m	33 (3.4)	6 (4.4)	39 (3.5)	6 (0.01)	8 (0.1)	14 (1.7)	4 (3.4)	2 (0.8)	6 ((4.2)	59 (2.9)
H.t	4 (0.1)	9 (6.6)	13 (1.2)	3 (0.004)	1 (0.01)	4 (0.01)	8 (6.8)	1 (0.04)	9 (6.3)	26 (0.2)
Total count	964 (47.1)	136 (6.6)	1100 (53.7)	723 (35.3)	81 (4)	804 (39.3)	118 (5.8)	25 (1.2)	143 (7)	2047 (100)

A. v: *Amblyomma variegatum*, A. c: *Amblyomma cohorense*, B. d: *Boophilus decoloratus*, R. e: *Rhipicephalus evertsi evertsi*, R. p: *Rhipicephalus praetextatus*, H.m: *Hyalomma marginatum*, H.t: *Hyalomma truncatum*.

### 3.5 Agroecological distribution and variations of tick species

**FIGURE 1:**
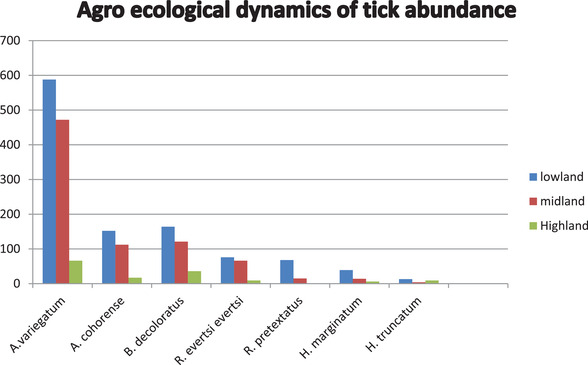
Variation of tick numbers collected on cattle according to the altitude

The result of the study prevailed that there was a highest significant (*p* < 0.05) difference in abundance and distribution of Ixodid tick species infesting cattle like *A. variegatum, A. cohorense, B. decoleratus, R. evertsi evertsi, R. praetextatus* and *H. marginatum* in lowland, highland and midland agroecological systems. Its distribution is highest in lowland and midland agroecological systems than highland agroecological systems. But in *H. truncatum* species, there was no significant (*p* > 0.05) difference in abundance and distribution in lowland, midland and highland agroecological systems (Figure 1 and Table 7).

**TABLE 7:** Comparison of risk of infestation by tick species in cattle‐based agroecological systems using Poisson regression analysis

Tick species	Agroecological systems	Count number	95% CI	*p* Value
A. V	Lowland	588	−0.84 to 0.67	0.000
	Midland	472		
	Highland	66		
A. C	Lowland	152	−0.96 to 0.62	0.000
	Midland	112		
	Highland	17		
B. d	Lowland	164	−0.78 to 0.49	0.000
	Midland	121		
	Highland	36		
R. e	Lowland	76	−0.94 to 0.50	0.000
	Midland	66		
	Highland	9		
R. p	Lowland	68	−2.30 to 1.32	0.000
	Midland	15		
	Highland	0		
H. m	Lowland	39	−1.34 to 0.57	0.000
	Midland	14		
	Highland	6		
H. t	Lowland	13	−0.71 to 0.24	0.339
	Midland	4		
	Highland	9		

A. v: *Amblyomma variegatum*, A. c: *Amblyomma cohorense*, B. d: *Boophilus decoloratus*, R. e: *Rhipicephalus evertsi evertsi*, R. p: *Rhipicephalus praetextatus*, H.m: *Hyalomma marginatum*, H. t: *Hyalomma truncatum*.

### 3.6 Seasonal variations of tick species

**FIGURE 2:**
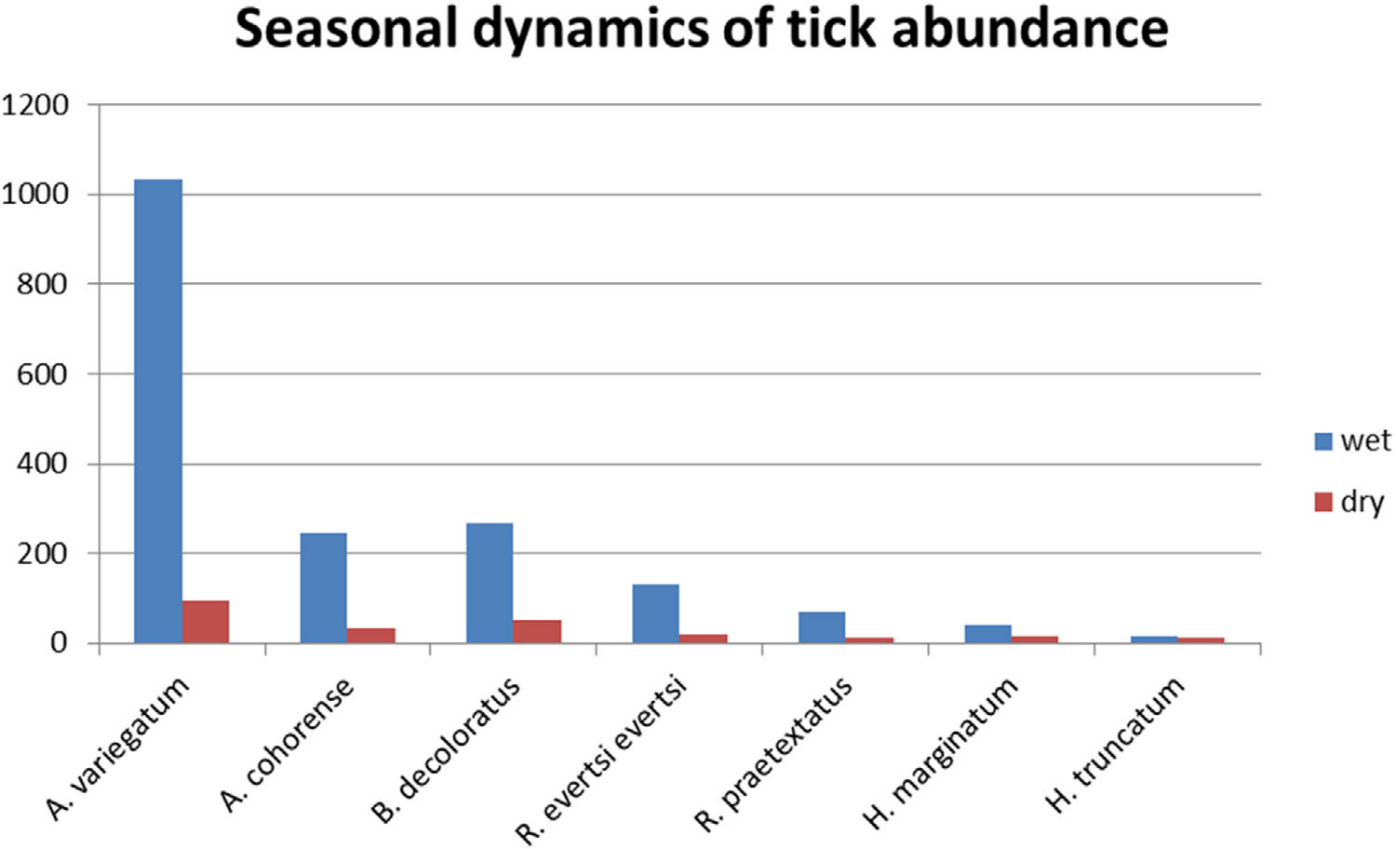
Variation of tick numbers collected on cattle according to the season

The current study indicated that there is a high difference in the prevalence of Ixodid tick infestation in season. Higher infestation was occurred in wet season than dry season. There was no seasonal variation in the occurrence of *H. truncatum* species (Figure 2).

## 4 DISCUSSION

The current study revealed that hard ticks are important external parasites and highly distributed in Ankasha and Jawi districts of Ethiopia. In the present study, a total of 2047 adult ticks were collected from 173 cattle with over all prevalence of 45% of tick infestation. This finding is in line with the previous report of 40.26% around Haramaya town (Yalew et al., 2017). Similarly, Kindalem (2015) reported 48.44% in and around Kombolcha. However, the current finding disagrees with Misgana (2017 who reported overall prevalence of 91.5% in Adaa and Boset districts Oromia regional state. The variation of these findings may be due to different management system, seasonal variation, agroecology, study design, different target animals.

The present study revealed that overall prevalence of 3% *B. bigemina* infection in cattle was recorded in Jawi district. The prevalence of *B. bigemina* in an area is the suggestion of the reflection of the distribution of its vectors, *Rhipicephalus evertsi* and *Rh. (Bo) decoloratus* both of which are present in Jawi District. This finding is in line with the previous finding of Bock et al. (2004). The current study indicated that the prevalence of infection in adult animals was higher than that of young cattle but there was no significant difference. This was in line with the report of Ayaz et al. (2013) from Pakistan. There was slightly higher infection in female cattle than male cattle in the study area. This finding is in line with the finding of Kocan et al. (2010) who found higher prevalence of babesiosis in female cattle.

In this study, the prevalence of tick infestation was significantly (*p* < 0.05) highest (56.2%) in poor conditioned cattle. This finding is in line with the previous reports performed by Amsalu et al. (2015) in Dangla, northwest Ethiopia with the prevalence of 62.9%. Because poor body conditioned animals had lower resistance to tick infestation and they exposed tick infestation during grazing on the field than medium and good body conditioned animals.

The variations in prevalence were higher in females than males but there was no statistical (*p* > 0.05) difference during the study period. It agrees with the report of Fentahun and Mohammed (2012) in Assosa, Ethiopia but it disagrees with the report of Zerihun and simeon (2013) in Benchimagi zone, Ethiopia. This might be due to the fact that most of the time males enter to feed lot and thus they have less accessibility to be infested with tick. Feedlot animals are most likely with reduced tick infestation since the environment is not suitable for the free‐living stages of tick (Jonsson, 2004). The sensitivity of thin blood smear is low and adequate for acute cases of *Babesia bovis* infection as compared to other techniques like IFAT, ELISA, complement fixation test and PCR (Salih et al., 2015). Blood smears are not reliable for detection of carrier animals. In these cases, molecular methods or serological diagnostic procedures are required demonstrate specific antibodies (Pohl, 2013). Polymerase chain reaction (PCR) assays can detect and differentiate *Babesia* species and are particularly useful in carriers than blood smear techniques (CFSPH, 2008). In the current study, *R. annulatus* and *R. microplus* were not recorded.

The rate of tick infestation was significantly (*p* = 0.04) higher in adult animals than young animals. This report coincides with the report Tadele et al. (2016) in Jabitennan woreda, West Gojjam, Ethiopia. The reason might be decreased contact of young animals with other groups of animals, which can be source of transmission. Most of the time many adult cattle graze in the pasture and forest, and the chance of getting tick infestation is increasing (Ramsi et al., 2007).

In the present investigation, there was a significant (*p* < 0.05) association between body condition of cattle with the risk of tick borne haemoparasite infection. This could be due to the fact that animals with poor body condition have lower immunity which encourages infection of animal by different organisms like babesia. Among the infected animals, three of them displayed a clinical sign of high weight loss and congested mucous membranes. The weight loss of infected animals might be related to anorexia and the rapidly dividing parasites in the red blood cells produce rapid destruction of the erythrocytes because the packed cell volume falls below 20%, which will lead to anaemia (Demessie & Derso, 2015). In addition, during this study, it was very common to see high burden of ticks in animal with poor body condition unlike those animals with good body conditions and this can increase rate of infection from babesia. This is in agreement with the previous reports of Bihonegn et al. (2015) in Benshangul Gumez Region, Western Ethiopia.

According to seasonal infection rate of bovine babesiosis, the seasonal prevalence of babesiosis infection using thin blood smears examination revealed the overall infection rate of cattle was recorded during the wet season. These results were similar with results of Kamani et al. (2010) who recorded that, the highest infection rate of Babesiosis was recorded in wet season and was low in dry season in cattle.

In the current study, *Amblyomma, Rhipecephalus, Subgenus Rhipecephalus (Boophilus) and Hyalomma* were the four tick genera identified. *Amblyomma* (68.7%) was the most abundant hard tick genus whereas *Hyalomma* (4.1%) was the least abundant genus among the identified genera in the study areas. This finding is in agreement with the report of the study done by Amante et al. (2014) in Diga, Western Ethiopia. *Hyalomma* was the least abundant tick species in the three agroecologies in the study areas with over all prevalence of 4.1%. This report coincides with the previous study with 3% prevalence by Hailu et al. (2016) in and around Bishoftu town, Oromia, Ethiopia. However, the current study is disagree with the study done by Fisseha and Mathe (2018) in Hossana district, Hadiya zone, Ethiopia stated that Hyalomma was the most abundant tick genus with the prevalence of 11.9%. This variation could be due to the difference of the season of tick collection and agroecological systems in study areas.

In the present study, the prevalence and abundance of hard tick infestation of cattle was highest in lowland and midland than highland. This finding is in line with previous results in Ethiopia reported by Mekonnen et al. (2007). It was due to the fact that lowland agroecological systems with high temperature and humidity are more suitable for tick multiplication and survival than in highland area as has been reported previous by Kumsa et al. (2012).

In the present investigation, tick abundance count was significantly (*p* = 0.000) higher in wet season than dry season. This report was in consistent with the report of Kumsa et al. (2012) that high humidity and temperature are crucial factors that influence the seasonal variation of ticks. However, this study is in disagreement with the study done by Kemal et al. (2016) who reported that there was no considerable difference in the prevalence of ticks within the wet and dry season. Additionally, this finding is in agreement with the study done by Mekonnen et al. (2007) that ticks were found on cattle throughout the study period, although higher tick counts were observed during the rainy than dry season. This might be attributed that the dry season results in lower relative humidity and higher environmental temperature, which influences the mortality of ticks due to desiccation (Mesele et al., 2010).

The finding of the present study indicated that *A. variegatum, A. cohorense, B. decoloratus, Rh. evertsi evertsi* and *Rh. pretextatus* are the most common encountered tick species in the study districts. On the other hand, *H. truncatum* and *H. margienatum* were collected in a small amount of quantity across the lowland, midland and highland agroecologies during the wet and dry climate. In this study, *A. variegatum* (55%) was the most prevalent tick species. This report coincides with a study done by Mekonnen (2017) who reports a prevalence of 57.3% in selected districts of North Gondar, Ethiopia. On the other hand, it was in disagreement with the outcome of the previous research done by Shiferaw (2005) in Wolayita, SNNP, Ethiopia who stated that *Boophilus decoloratus* was the most abundant tick species. The observation of high tick counts in lowland agroecological systems on cattle in the present study is most probably attributed to the vast and seasonal availability of grazing land and unrestricted cattle movement from place to place in the lowland than in both the midland and highland agroecological zones (Rahmeto et al., 2010).

*Amblyomma variegatum* was significantly (*p* = 0.000) higher in count during the wet season than dry season. It agrees with the previous study reports of Misgana (2017) in central Ethiopia. The finding of the current study is in line with other African countries researchers like reports done by Salih et al. (2008) from Southern Sudan and Walker and Koney (1999) from Ghana reported that *A. variegatum* was abundant during the rainy season. It is attributed to the fact that activation and reproduction of quiescent adult are initiated by rainfall.

*Boophilus decoloratus* was the second observable tick species in the study areas with the prevalence of 15.7%. This finding coincides with the previous reports of Gedilu et al. (2014) and Bedasso et al. (2014) who reported *Rh*. (*Bo.) decoloratus* as the second prevalent tick species. But it disagrees with the previous report done by Getachew et al. (2014) that *B. decoloratus* was the most abundant tick species in Dembia, North Gondar, Ethiopia with the prevalence of 40%. This finding report is also in agreement with the report done by Mattioli et al. (1997) on *B. decoloratus* as the second most abundant tick in Gambia. This difference might be attributed to the differences in climates and altitude among the study areas.

*Amblyomma cohorense* was the third most abundant tick species in the study areas with a prevalence of 17%. This finding is consistent with the previous research done by Mekonnen (2017) in North Gondar, Ethiopia. Amante et al. (2014) also reported that 18% prevalence of *Am. cohorense* in Diga, Ethiopia as the third more abundant tick species. On the contrary, the finding of the current study was in disagreement with the previous report of Bedaso et al. (2014) done in Haramaya, Ethiopia.

*Rhipecephalus evertsi everttsi* was the fourth abundant tick recorded in the study areas during the wet and dry seasons with a prevalence of 7.4%. This report of finding coincides with the previous reports of study performed by Fentahun and Mohammed (2012). It was also consistent with the report of Tamirat et al. (2015) in Bedele, Ethiopia. But it was in disagreement with the previous report of Huruma et al. (2015) with the prevalence of 53.4%, which was done in Sebeta, Ethiopia.

In the present investigation, *H. truncatum* was the least abundant tick recorded in the study areas with the prevalence of 0.2%, which is in agreement with the previous report of Walker and Koney (1999) in Ghana. It is also in line with the report of Biru et al. (2017) who reported that *H. truncatum* is the least abundant tick species in Harerge, southeast Ethiopia. Based on the finding of the current study, the lowest number of *T. truncatum* was recorded in all agroecologies and during the wet and dry season with nonsignificance (*p* > 0.05) variations.

## 5 CONCLUSION

The current study revealed that there was high prevalence of Ixodid tick infestation and tick borne haemoparasite. A total of five potential risk factors were evaluated to determine the association with tick infestation and tick borne haemoparasite. Age, body condition, agroecological systems and season were significantly associated with tick infestation whereas body condition and season were significantly associated with the risk of tick borne haemoparasite. In this study, a total of four genera and seven tick species were identified. Of them *A. variegatum* and *B. decoloratus* were the predominant species encountered. From this study result, it is possible to conclude that agroecological systems and season play great role in the variation in distribution and abundance of tick species and dynamics. Tick infestation of cattle was significantly higher in lowland than midland and highland. The tick burden was also higher in wet period than dry period. The total tick burden was also higher in wet period compared to dry period. This high abundance of tick infestation of cattle and their tick borne haemoparasite in the study areas require high attention at all concerned levels to minimise the impacts on the health and productivity of cattle to improve the living standards of farmers of the study areas. Therefore, strategically and effective tick control programs depending on the distribution pattern of ticks and factors responsible for their distribution should be implemented. Likewise, further research using molecular diagnostic techniques should be conducted for investigation of tick‐borne haemopathogens on livestock in Ethiopia.

## CONFLICT OF INTEREST

The other authors declare no conflict of interest.

## FUNDING

No funding was obtained for this study.

## ETHICS STATEMENT

The authors confirm that the ethical policies of the journal, as noted on the journal's author guidelines page, have been adhered to. No ethical approval was required, as this study required no animal experiments.

## AUTHOR CONTRIBUTIONS

**Hailemariam Adugna**: conceptualisation, data curation, investigation and methodology writing‐original draft preparation. **Habtamu Tamrat**: Methodology, writing‐original draft preparation, formal analysis, writing – review and editing, supervision, validation.

### PEER REVIEW

The peer review history for this article is available at https://publons.com/publon/10.1002/vms3.878.

## Data Availability

The data that support the findings of this study are available from the corresponding author upon reasonable request.
